# An acquired stable variant of a dicentric dic(9;20) and complex karyotype in a Syrian childhood B-acute lymphoblastic leukemia case

**DOI:** 10.1186/s13039-020-00499-x

**Published:** 2020-07-10

**Authors:** Abdulsamad Wafa, Rami A. Jarjour, Abdulmunim Aljapawe, Suher ALmedania, Thomas Liehr, Joana B. Melo, Isabel M. Carreira, Moneeb A. K. Othman, Walid Al-Achkar

**Affiliations:** 1 Department of Molecular Biology and Biotechnology, Human Genetics Division, Atomic Energy Commission, Damascus, Syria; 2 Department of Molecular Biology and Biotechnology, Mammalians Biology Division, Atomic Energy Commission, Damascus, Syria; 3 Jena University Hospital, Institute of Human Genetics, Jena, Germany; 4 Cytogenetics and Genomics Laboratory, Faculty of Medicine, University of Coimbra, Coimbra, Portugal; 5 CIMAGO-Center of Investigation On Environment Genetics and Oncobiology-Faculty of Medicine, University of Coimbra, Coimbra, Portugal

**Keywords:** Acute lymphoblastic leukemia, Complex karyotype, Dicentric dic(9;20), Array-based multicolor banding (aMCB), Array comparative genomic hybridization (aCGH), Prognostic factors

## Abstract

**Background:**

About 25 years ago, the acquired chromosome abnormality dicentric dic(9;20)(p11 ~ 13;q11) was seen described as a non-random aberration in B-cell precursor acute lymphoblastic leukemia (BCP-ALL). Yet, about 200 cases were reported. However, dicentric dic(9;20) is a subtle abnormality which easily may be mixed up with monosomy 20 and/or del(9p). The dicentric dic(9;20) can be found as a sole chromosomal abnormality or can be masked within complex rearrangements; also, a dicentric dic(9;20) is often associated with mono- or biallelic loss of *CDKN2A* gene.

**Case presentation:**

Here we report a case of 16-year-old male diagnosed with a de novo pre-B-ALL. Molecular approaches (array-based multicolor banding (aMCB) and array comparative genomic hybridization (aCGH)) were applied, and a unique complex karyotype involving six chromosomes was identified. It included three previously unreported chromosomal aberrations: dicentric dic(9;20;X), deletion del(7)(p22.2p15.2) and dicentric dic(7;13). The dicentric dic(9;20;X) also led to monoallelic loss of tumor suppressor gene *CDKN2A*. After successful chemotherapeutic treatment the patient experienced a relapse with a secondary ALL without complex karyotype but a deletion del(19)(p13). Unfortunately, the patient died after 17 months of the initial diagnosis.

**Conclusions:**

To the best of our knowledge, a comparable childhood ALL associated with such complex karyotype and deletion del(19)(p13) in secondary ALL was not previously reported. Thus, the complex karyotype with dicentrc dic(9;20;X) seems to indicate for a poor prognosis.

## Background

The stable chromosome abnormality dicentric dic(9;20)(p11 ~ 13;q11) was first reported as a non-random aberration in B-cell precursor acute lymphoblastic leukemia (BCP-ALL) in 1995 [1, 2]. Even though dicentric dic(9;20) can easily by missed and/or mixed up with other rearrangements (like monosomy 20 and/or del(9p)) in banding cytogenetics, still, already 199 cases have been published [1–5].

The dicentric dic(9;20) is more common in pediatric ALLs (2%) than in adult cases (< 1%) and seems to be more frequent in females [3]. The median age at diagnosis is 3 years; the median leucocyte count is 20–30 × 10^9^/l [6]; an event-free survival (EFS) and overall survival (OS) up to 5 years are reached by 62 and 82% of the patients, respectively. Accordingly, relapse cases are quite common and post-relapse treatment of many patients was successful [7].

All BCB-ALL cases reported had an immunophenotypes showing positive results for TdT, HLA-DR, CD10, CD19 and CD24, and negative for myeloid markers [1, 2, 4, 5]. The prognostic impact of dicentric dic(9;20) is still unclear, but most reported patients have attained complete remission; thus, such patients are suggested to have a good prognoses [1, 2, 4, 5]. Interestingly, unrecognized dicentric dic(9;20) cases may also be included in cases with monosomy 20 as sole abnormality in ALL; thus, it is noteworthy that the latter is considered to be a favorable prognostic marker [8, 9].

Dicentric dic(9;20) can occur as a sole cytogenetic abnormality, or in the context of a more complex karyotype [7]. Common additional genetic changes in ALL with dicentric dic(9;20) are deletions involving chromosome 13q and gains of chromosomes X, 8 and 20 [3, 4, 7]. Based on data obtained by fluorescence in situ hybridization (FISH) it is known that dicentric dic(9;20) can occur in the presence of the *BCR-ABL1* and *ETV6-RUNX1* fusion genes [7]. Furthermore, for the *CDKN2A* (cyclin-dependent kinase inhibitor 2A) gene in 9p21, mono- or biallelic deletions were also repeatedly seen [10, 11].

We present here clinical, cytogenetic and molecular data of bone marrow cells obtained from a de novo childhood pre-B-ALL case with a complex karyotype and relapse, involving a variant dicentric dic(9;20).

## Case presentation

On 30 Jun 2016, a 16-year-old male patient without any known medical background presented with a 1 month history of fatigue and fever without sweating. He had no familial history of malignancies and no social and environmental history or exposure to toxins and animals. Initial laboratory evaluation of peripheral blood (PB) revealed white blood cells (WBC) of 52.2 × 10^9^/l (88% were blasts). He was treated with Predlon 60 mg/day per 10 days. Afterwards, physical examination and ultrasound at our hospital showed no splenomegaly, however, several lymphadenopathies (sternocleidomastoidal (1 cm) and right of subaxilla (1 cm)), normal heart rate (90/min) and his blood pressure was 12/6. His PB showed: WBC 3.5 × 10^9^/l (neutrophils 33%, lymphocytes 64%), Hb = 7.5 g/dl, and platelets = 49.4 × 10^9^/l. Serum biochemistry analyses were: Calcium (Ca^+ 2^) 9.9 mmol/l (normal value 8.5–10.3); LDH 229 U/l (normal level < 460); β2-microglbulin 3.32 mg/l (normal value 0.61–3.7); alanine aminotransferase level was 24 U/l (normal up to 40 U/l); aspartate aminotransferase level 17 U/l (normal up to 40 U/l); creatinine was 0.57 μmol/l (normal 45–120); Urea 38 mmol/l (normal 10–50); Sodium (Na^+^) 137 mmol/l (normal 135–148), Potasium (K^+^) 4.7 mmol/l (3.5–5.2), total protein 6.2 g/dl (normal 6.6–8.7), albumin 4.2 g/dl (normal 3.8–5.4). Bone marrow (BM) aspiration revealed hypercellularity with 90% of lymphoblasts. In cerebrospinal fluid aspiration no cells were found.

He was diagnosed as having pre-B-ALL according to the World Health Organization (WHO) classification. Thus, the patient was treated further according to GRALL 2003 chemotherapy protocol. Two days after initiating GRALL 2003 chemotherapy, the patient developed neutropenia, was given Neupogen and restarted chemotherapy protocol. The patient suffered from neutropenia and fever many times during chemotherapy. All chromosomal aberrations were vanishing during the chemotherapeutic treatment. After 17 months of treatment the patient relapsed. BM aspiration revealed 10% of lymphoblasts and PB showed: WBC 1.7 × 10^9^/l (neutrophils 60.5%, lymphocytes 32.2%, and immature cells 7.3%); Hb = 13.6 g/dl; and platelets = 216 × 10^9^/l. The patient received cytosar 3.5 g (twice per day for 4 days) and doxorubcin 50 mg/m^2^ for 3 days and a wide spectrum of antibodies.

Approximately 2 months after relapse patient died due to respiratory and heart arrest, as well as neutropenia. No autopsy was performed. Patient’s father agreed with scientific evaluation of his case and the study was approved by the ethical committee of the Atomic Energy Commission, Damascus, Syria.

## Results

**Fig. 1:**
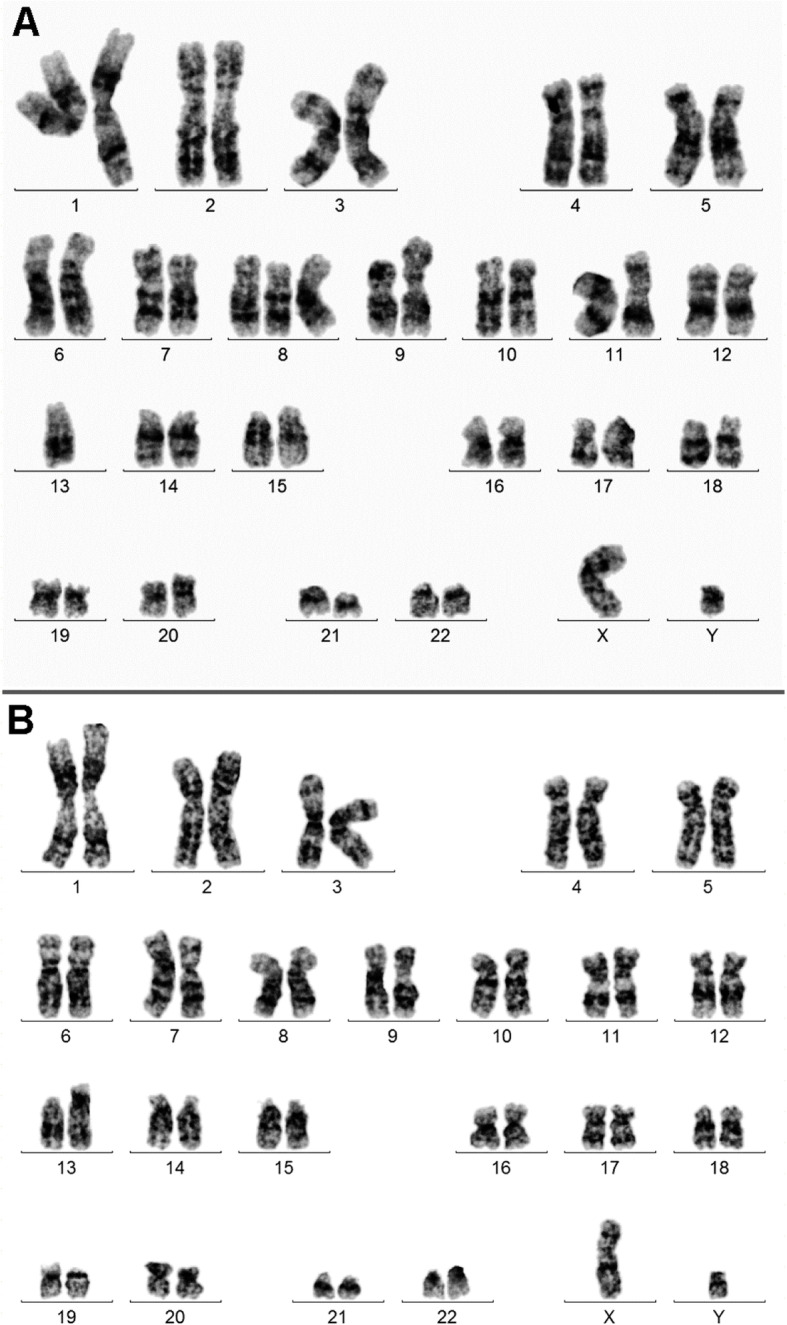
GTG-banding revealed a complex karyotype in BCP-ALL (**a**), and a karyotype 46,XY,del(19)(p13) after relapse to secondary ALL (**b**)

**Fig. 2:**
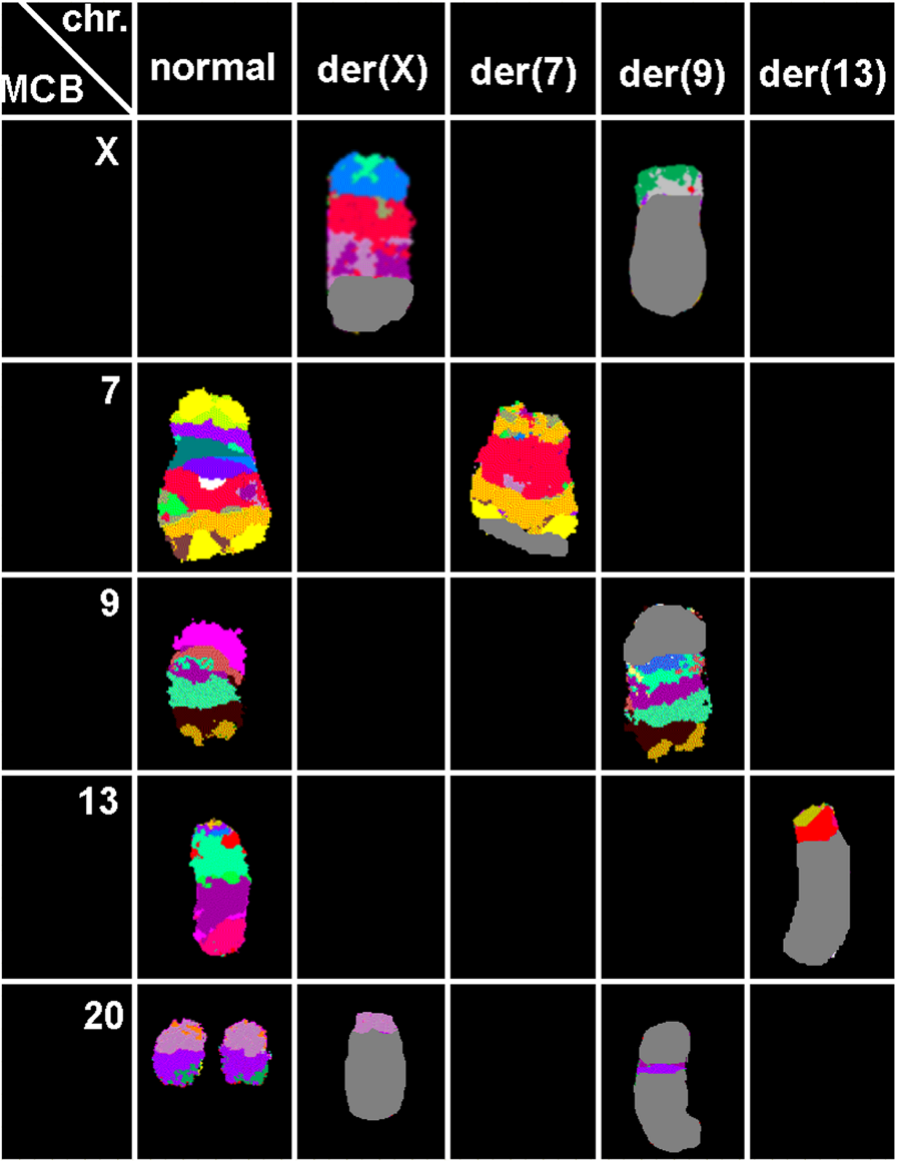
aMCB results are shown. The normal chromosomes are depicted on the left side and the derivative of the corresponding chromosomes on the right side of normal chromosomes. The unstained regions when using chromosome-specific aMCB-probe sets on the derivative chromosomes are shown in gray. Der = derivative chromosome

GTG-banding was performed on BM sample according to standard procedures [12] prior and post chemotherapy. A minimum of 20 metaphase cells derived from unstimulated BM culture were analyzed. Karyotypes were classified according to the International System for Human Cytogenomic Nomenclature [13]. Prior to chemotherapy treatment GTG-banding revealed a karyotype 46,XY,der(X)t(X;?)(?;?),t(7;?)(?;?),+8,dic(9;?)(?;?),-13[9]/47,XY,der(X)t(X;?)(?;?),+8,dic(9;?)(?;?)[8]/46,XY[3] (Fig. 1a). Further FISH analysis including home-made whole chromosome painting (WCP) probes for chromosomes 1, 2, 3, 5, 6, 7, 8, 9, 10, 13, 15, 16, 17, 19, 20, 21, 22 and X and array-based multicolor banding (aMCB) probes for chromosomes 7, 9, 13, 20 and X were done as previously reported (results are shown in Fig. 2) [14].

**Fig. 3:**
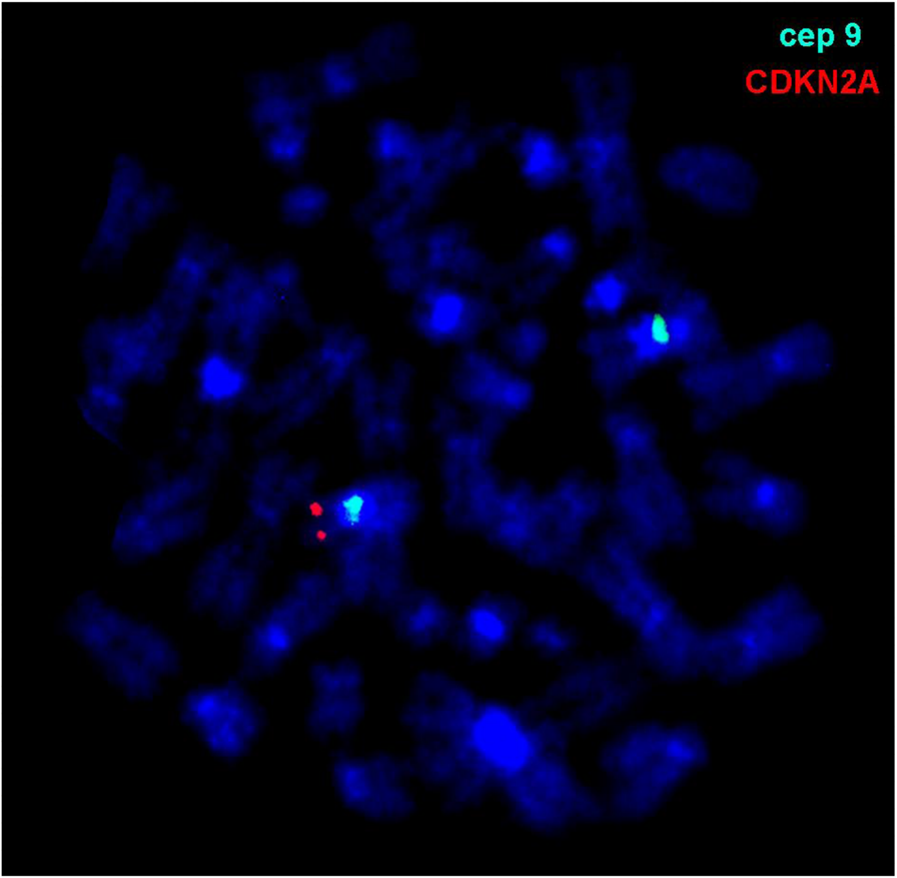
FISH result of *CDKN2A* showed monoallelic deletion on the der(9)(Xpter->Xp21::20p12->q11.2::9p13.2->9qter)

Besides, commercially available probes were applied: Zyto*Light*®SPEC CDKN2A/CEN9 (in 9p21.3 and 9p11q11 dual color probe) (Fig. 3) and Zyto*Light*®SPEC JAZF1 (7p15.2p15.1 Break Apart Probe) all from ZytoVision GmbH (Bremerhaven, Germany), LSI ETV6 (in 12p13.2 dual color break part probe) and LSI p53/ATM (in 17p13.1 and 11q22.3 dual color probe) all from Vysis (Abbott GmbH & Company, KG, Wiesbaden, Germany). A total of 10 metaphase spreads were analyzed, each, and (where applicable) 200 interphase nuclei were examined, using a fluorescence microscope (AxioImager.Z1 mot, Zeiss) equipped with appropriate filter sets to discriminate between a maximum of five fluorochromes and the counterstain DAPI (Diaminophenylindol). Image capturing and processing were carried out using an ISIS imaging system (MetaSystems, Altlussheim, Germany).

The final karyotype prior to chemotherapeutic was finally defined as:

46,XY,der(X)t(X;20)(p21;p12),der(7)dic(7;13)(p15.2q12.3),+8,der(9)(Xpter->Xp21::20p12->q11.2::9p13.2->9qter),-13[9]/46,XY[3].

Genomic DNA was extracted from BM cells prior to chemotherapy treatment and aCGH was performed using the Agilent Sure Print G3 Human Genome Microarray 180 K as previously described [14].

Array-CGH revealed four losses of copy numbers in:

- 7p22.3 to 7p15.2 at positions 109,626 to 26,260,755 including five COSMIC census cancer genes;
- 7p14.2 to 7p11.2 at positions 35,292,065 to 56,174,888 including 3 COSMIC census cancer genes;
- 9p24.3 to 9p13.2 at positions 207,437 to 37,270,400 including 10 COSMIC census cancer genes, and
- 13q12.3 to 13q24 at positions 32,035,219 to 115, 059,020 including 10 COSMIC census cancer genes.

Besides, four gain of gains of copy numbers were identified by array-CGH in:

- whole chromosome 8, including 34 COSMIC census cancer genes;
- 20p13 to p11.1 at positions 60,747 to 25,713,574 including 2 COSMIC census cancer genes;
- 20q11.2 to 20q11.2 at positions 29,467,937 to 29,948,374 (no COSMIC census cancer gene identified), and
- 20q13.13 to 20q13.13 at positions 46,828,431 to 48,880,347 (no COSMIC census cancer gene identified) (Tab. 1).

**Table 1:** Summary of CNAs detected by aCGH

Chr.	Start –End band	Genomic position: start- end GRCh37/hg19	Variant type	Size (Mb)	COSMIC census cancer gene(s) within the region
7	p22.3p15.2	109,626-26,260,755	loss	26.1	***CARD11, PMS2, RAC1, MACC1, HNRNPA2B1***
	p14.2p11.2	35,292,065-56,174,888	loss	20.8	***SFRP4, IKZF1, EGFR***
8	p23.3p11.1	176,452-43,399,198	gain	43.2	***ARHGEF10, PCM1, LEPROTL1, WRN, NRG1, NSD3, FGFR1, ANK1, KAT6A, IKBKB, HOOK3***
	q11.1q24.3	46,939,154-146,294,098	gain	99.3	***TCEA1, PLAG1, CHCHD7, PREX2, NCOA2, HEY1, CNBD1, NBN, RUNX1T1, CDH17, COX6C, PABPC1, UBR5, EIF3E, RSPO2, CSMD3, RAD21, EXT1, MYC, NDRG1, FAM135B, RECQL4***
9	p24.3p13.2	207,437-37,270,400	loss	37.1	***JAK2, CD274, PDCD1LG2, PTPRD, NFIB, PSIP1, MLLT3, CDKN2A, FANCG, PAX5***
13	q12.3q24	32,035,219-115,059,020	loss	83.0	***BRCA2, NBEA, LHFPL6, FOXO1, LCP1, RB1, CYSLTR2, GPC5, SOX21, ERCC5,***
20	p13p11.1	60,747-25,713,574	gain	25.6	***SIRPA, CRNKL1,***
	q11.2	29,467,937-29,948,374	gain	0.5	n.a.
	q13.13	46,828,431-48,880,347	gain	2.05	n.a.

Immunophenotyping was performed on BM specimen prior to chemotherapy treatment using a general panel of antibodies against antigens specific for different blood cell lineages and blood cell types [15]. Those antibodies were against: CD1a, CD2, CD3, CD4, CD5, CD8, CD10, CD11b, CD11c, CD13, CD14, CD15, CD16, CD19, CD20, CD22, CD23, CD32, CD33, CD34, CD36, CD38, CD41a, CD45, CD56, CD57, CD64, CD79a, CD103, CD117, CD123, CD138, CD209, CD235a and CD243; In addition to antibodies to Kappa and Lambda light Chains, sIgD, sIgM, and HLADr. All antibodies were from BD Biosciences. Samples analyzed on a BD FACSCalibur™ flow cytometer. Auto fluorescence, viability, and isotype controls were included. Flow cytometric data acquisition and analysis conducted by BD Cellquest™ Pro software. Interpretations of FCM results were according to [16].

FCM analysis of BM specimen prior to chemotherapy treatment characterized this case as Pre-B-ALL according to WHO classifications. The abnormal cell population (51%) was positive for CD45^dim^, CD34, HLADr, CD19, CD10, cCD79a, and expressed CD13 and CD33 heterogeneously. Blast cell population was negative for CD3, CD117, CD14, CD64, CD7, CD2 and CD5.

After chemotherapy and relapse GTG-banding revealed a karyotype of 46,XY[18],46,XY,del(19)(p13)[2] (Fig. 1b).

## Discussion and conclusions

According to the literature, the dicentric dic(9;20) has been reported in 199 ALL cases listed in Mitelman database [3]. Dicentric dic(9;20) with trisomy of chromosomes 8 or 21 were seen in 10 and 7 ALL cases, respectively [3]. A translocation t(X;9) involving short and/or long arms of these chromosomes has been found in 11 ALL cases [3]. In addition, partial deletion of the short arm of chromosome 7 [del(7)(p14p11)], and derivative del(19)(p13) were previously reported in 2 and 102 ALL cases, respectively [3]. Interestingly, translocation t(X;20)(p21;p12), derivative del(7)(p22p15), dicentric dic(7;13) have never been described in ALL cases. To the best of our knowledge, a combination of all these complex rearrangements with new formation of dicentric dic(9;20) in one ALL case at diagnosis was not previous reported yet [3].

The dicentric dic(9;20) contains centromeres of both chromosomes 9 and 20, resulting in loss of 9p and 20q material [1, 2, 4, 5], which occurs at a low frequency in ALL cases (2% in children and < 1% in adult ALL patients), predominantly in females [3; 7].

The dicentric dic(9;20) can be found as a sole chromosomal aberration (~ 40% of the ALL cases) or with additional chromosomal aberrations (ACAs) (60% of the ALL cases) [17]. Strefford et al. [11] have suggested that the dicentric dic(9;20) is not associated with a recurrent gene rearrangement. While Coyaud et al. [18] noted that dicentric cases can masking a complex rearrangement. Our present case represents a novel formation of dic(9;20) with loss 9p and 20q in a chromosomal aberration involving X-chromosome.

Notably, the dicentric dic(9;20)-positive leukemia is frequently associated with hetero- or homozygous loss of *CDKN2A* gene in 31% of all cases analyzed by FISH [17]. However, whether loss of function of this gene is pathogenetically and/or clinically important in dicentric dic(9;20)-positive ALL, remains to be elucidated, but is most likely valid [17]. Other common ACAs included gains of X and 21, both of which are frequent in other subtypes of BCP-ALL [6].

A complex karyotype has been generally classified as ≥3 unrelated chromosomal abnormalities in ALL cases with the absence of established translocations (t[9;22], t[v;11q23], t[1;19], t[8;14], and t[14q32]) [19]. Moorman et al. [19] demonstrated that those ALL patients with complex karyotype ≥4 or more unrelated chromosomal abnormalities had a poor outcome in terms of OS and EFS, with most of the relapses occurring in the first 2 years after diagnosis. While, Motll’o et al. [20] showed that a complex karyotype was not associated with adverse prognosis in adult ALL patients treated with risk-adapted or subtype-oriented protocols.

In conclusion, we report the first pre-B-ALL case obtained complex karyotype with a new acquired stable variant of a dicentric dic(9;20) resulting from masked partial trisomy 20. In addition, monoallelic deletion of tumor suppressor gene *CDKN2A* and subsequent deletion del(19p13) without all the previously observed changes in the secondary ALL were seen. Overall, such complex chromosomal changes seem to have adverse prognosis in pre-B-ALL.

## Data Availability

All relevant data and material is included in this publication.

## Abbreviations

ACAs: Additional chromosomal aberrations
aCGH: array comparative genomic hybridization
aMCB: array-proven multicolor banding
ALL: Acute lymphoblastic leukemia
BM: Bone marrow
DAPI: 4′,6- diamino-2-phenylindole
EFS: Event-free survival
FISH: Fluorescence in situ hybridization
HGB: Hemoglobin level
OS: Overall survival
PB: Peripheral blood
WBC: White blood cells
WCP: Whole chromosome painting
WHO: World health organization
